# Supplementary material for: Amyloid-Beta Co-Pathology Is a Major Determinant of the Elevated Plasma GFAP Values in Amyotrophic Lateral Sclerosis
Source: Int J Mol Sci. 2023 Sep 12;24(18):13976. doi: 10.3390/ijms241813976 (PMC10531493; doi:10.3390/ijms241813976)
Supplement: Supplementary file 1 [file ijms-24-13976-s001.zip › ijms-2544943-supplementary.pdf]

Amyloid-beta co-pathology is a major determinant of the elevated plasma GFAP values in amyotrophic lateral sclerosis

Andrea Mastrangelo†, Veria Vacchiano†, Corrado Zenesini, Edoardo Ruggeri, Simone Baiardi, Arianna Cherici, Patrizia Avoni, Barbara Polischi, Francesca Santoro, Sabina Capellari, Rocco Liguori, Piero Parchi.

[a] Dipartimento di Scienze Biomediche e Neuromotorie, Università di Bologna, (DIBINEM), Bologna, Italia

[b.] IRCCS, Istituto delle Scienze Neurologiche di Bologna, Bologna, Italia

†: These authors contributed equally to this work and share first authorship.

Correspondence to: Prof. Piero Parchi, IRCCS Istituto delle Scienze Neurologiche, Ospedale Bellaria, Via Altura 1/8, 40139 Bologna, Italy. E-mail: piero.parchi@unibo.it. Phone: +39-051-4966740. Fax: +39-051-4966208.

Keywords: amyotrophic lateral sclerosis, Alzheimer’s disease, GFAP, biomarker

SUPPLEMENTARY RESULTS

Table S1. Neuropsychological results in the whole ALS cohorts and in the AD/ALS and not-AD/ALS subcohorts

	Total ALS (n=156)	AD/ALS (n=9)	not-AD/ALS (n=147)
**ECAS total scores** (age-and education-adjusted), **n**	108.2 (96.1-116.8),88	89.2 (79.9-112.0),4	108.4 (96.8-116.8),84
**ECAS total scores** (equivalent scores), **n**	4.0 (3.0-4.0),88	2.0 (1.2-3.5),4	4.0 (3.0-4.0),84
**ECAS ALS-specific scores** (age-and education-adjusted), **n**	79.7 (71.9-86.1),88	64.4 (61.2-84.4),4	79.9 (72.6-86.1),84
**ECAS ALS-specific scores** (equivalent scores), **n**	4.0 (3.0-4.0),88	2.0 (2.0-3.5),4	4.0 (3.0-4.0),84
**ECAS language sub-scores** (age-and education-adjusted), **n**	25.5 (23.2-27.0),87	22.8 (20.4-28.0),3	25.6 (23.3-27.0),84
**ECAS verbal fluency sub-scores** (age-and education-adjusted), **n**	18.7 (15.2-21.1),86	18.3 (15.2-21.2),3	18.7 (15.0-21.1),83
**ECAS executive functions sub-scores** (age-and education-adjusted), **n**	36.3 (32.1-40,1),87	29.3 (22.9-40.3)	36.7 (32.4-40.1),84
**ECAS ALS-nonspecific scores** (age-and education-adjusted), **n**	27.3 (23.9-30.6),88	24.8 (18.4-27.8),4	27.3 (24.1-30.6),84
**ECAS ALS-nonspecific scores** (equivalent scores), **n**	4.0 (2.0-4.0),88	3.0 (1.2-4.0),4	4.0 (2.0-4.0),84
**ECAS memory sub-scores** (age-and education-adjusted), **n**	16.0 (13.0-18.44),87	15.7 (9.1-17.1),3	16.0 (13.0-18.6),84
**ECAS visuo-spatial sub-scores** (age-and education-adjusted), **n**	11.9 (11.4-12.2),87	11.8 (10.9-12.5),3	11.9 (11.4-12.2),84
**MMSE, n**	29.0 (28.0-30.0),114	28.0 (22.5-29.0),5	29.0 (28.0-30.0),109
**MMSE** (age-and education-adjusted), **n**	28.1 (26.7-29.0),113	25.7 (22.9-29.4),5	28.8 (27.5-30),108
**Rey Auditory Verbal Learning test** (immediate recall), **n***	40.2 (32.9-46.3),114	34.6 (21.1-40.4),5	40.3 (33.0-46.3),109
**Rey Auditory Verbal Learning test** (delayed recall), **n***	8.0 (5.9-9.7),113	9.4 (2.7-10.5),5	7.9 (6.0-9.7),108
**Short-term visual memory, n***	19.6 (17.2-20.9),114	15.8 (14.2-18.0),5	19.7 (17.4-20.9),109
**Simple verbal analogies test, n***	16.3 (13.7-17.6),114	15.7 (13.4-17.3),5	16.4 (13.7-17.7),109
**BDMB, n***	1.8 (1.1-2.3),107	1.1 (-0.5-2.1),5	1.8 (1.2-2.3),102
**FAB, n***	16.4 (14.6-17.4),27	16.9 (16.4-17.4),2	16.0 (14.5-17.6),25
**Freehand copy of drawings test, n***	10.4 (9.5-11.4),96	9.5 (7.2-10.0),4	10.6 (9.5-11.5),92
**Category fluency test, n***	38.0 (31.0-46.0),111	29.3 (18.2-39.9),4	38.0 (31.2-46.0),107
**Letter fluency test, n***	30.3 (22.4-37.4),110	23.0 (14.3-33.9),4	30.4 (23.9-37.6),106

*Test scores are age- and education-adjusted;
Test scores are expressed as median (interquartile range).

Abbreviations: AD, Alzheimer's Disease; ALS, amyotrophic lateral sclerosis; BMDB, Brief Mental Deterioration Battery; ECAS, Edinburgh Cognitive and Behavioural ALS Screen; FAB, Frontal assessment Battery; MMSE, Mini Mental State Examination.

Table S2. Univariable Cox Regression analysis for prognostic value of plasma biomarkers and clinical-laboratory variables in ALS patients

**Variable**		**Univariable COX regression**	
		**HR (95% CI)**	**p-value**
**Sex**		1.34 (0.84-2.15)	0.224
**Age at sampling**		1.03 (1.009-1.05)	**0.005**
**Time from clinical onset to sample**		0.87 (0.72-1.05)	0.143
**King's stage**	**Stage 1**	Ref	**<0.001**
	**Stage 2**	1.88 (0.25-14.3)	0.543
	**Stage 3**	4.78 (0.66-34.59)	0.122
	**Stage 4**	32.99 (4.07-267.56)	**0.001**
**BMI**		0.99 (0.94-1.04)	0.617
**DPR**	**Slow progressors**	Ref	Ref
	**Intermediate progressors**	2.27 (1.31-3.94)	**0.004**
	**Fast progressor**	4.15 (2.37-7.27)	**<0.001**
**FTD status**		1.97 (1.01-3.85)	**0.048**
**Onset type**	**Spinal**	Ref	Ref
	**Bulbar**	2.34 (1.39-3.94)	**0.001**
	**Pyramidal**	1.13 (0.35-3.64)	0.837
	**Pseudopolyneuritic**	0.84 (0.33-2.12)	0.716
**Clinical Phenotype**	**Classic**	Ref	
	**Bulbar**	1.76 (0.96-3.21)	0.067
	**PUMN**	1.03 (0.53-1.99)	0.938
	**PLMN**	1.09 (0.46-2.56)	0.843
**ALSFRS-R scale**		0.89 (0.86-0.92)	**<0.001**
**Genetic status**	**Wild type**	Ref	Ref
	SOD1	1.35 (0.33-5.52)	0.678
	C9Orf72	1.59 (0.81-3.1)	0.179
**Blood-brain barrier index**		1.02 (0.96-1.08)	0.58
**CPK levels**		0.99 (0.99-1)	0.183
**Plasma GFAP**		2.46 (1,91-3,01)	**<0.001**
**Plasma GFAP**	**Lowest tertile**	Ref	Ref
	**Intermediate tertile**	1.21 (0.68-2.13)	0.519
	**Highest tertile**	2.80 (1.59-4.92)	**<0.001**
**Plasma p-Tau181**		1.11 (1.02-1.2)	**0.016**
**Plasma NfL**		1.01 (1.006-1.01)	**<0.001**

Significant p-values are reported in bold.

Abbreviations: ALSFRS-R, amyotrophic lateral sclerosis functional rating scale revised; BMI, body mass index; CI, confidence interval; CPK, creatine phosphokinase; DPR, disease progression rate; FTD, frontotemporal dementia; GFAP, glial fibrillary acidic protein; HR, hazard ratio; NfL, neurofilament light chain; PLMN, prevalent lower motor neuron; p-tau181, phosphorylated tau 181; PUMN, prevalent upper motor neuron; Ref, reference.

Table S3. Multivariable Cox Regression analysis for plasma GFAP and clinical prognostic factors in ALS

Variable		HR (95% CI)	P-value
**Plasma GFAP**	**Lowest tertile**	Ref	Ref
	**Intermediate tertile**	0.83 (0.43-1.60)	0.58
	**Highest tertile**	0.96 (0.44-2.08)	0.92
**Age at sampling**		1.02 (0.99-1.05)	0.06
**Onset type**	**Spinal**	Ref	Ref
	**Bulbar**	2.23 (1.24-4.02)	**0.007**
	**Pyramidal**	0.48 (0.13-1.80)	0.28
	**Pseudopolyneuritic**	0.45 (0.17-1.22)	0.12
**ALSFRS-R scale**		0.91 (0.87-0.95)	**<0.001**
**FTD status**		1.22 (0.58-2.58)	0.60
**DPR**	**Slow progressors**	Ref	Ref
	**Intermediate progressors**	2.33 (1.27-4.24)	**0.006**
	**Fast progressors**	2.58 (1.36-4.89)	**0.004**

Significant p-values are reported in bold.
Abbreviations: ALSFRS-R, amyotrophic lateral sclerosis functional rating scale revised; CI, confidence interval; DPR, disease progression rate; FTD, frontotemporal dementia; GFAP, glial fibrillary acidic protein; HR, hazard ratio; Ref, reference.
